# Chronological changes in strawberry tongue in toxic shock syndrome toxin‐1–mediated Exanthematous Disease

**DOI:** 10.1002/jgf2.376

**Published:** 2020-09-20

**Authors:** Akihiko Shimizu, Yukako Ebara, Shigeru Nomura, Yoshiyuki Yamada

**Affiliations:** ^1^ Department of Allergy Infectious Diseases and Immunology Gunma Children's Medical Center Shibukawa Japan

**Keywords:** *Staphylococcus aureus*, strawberry tongue, toxic shock syndrome toxin‐1–mediated exanthematous disease

## Abstract

Strawberry tongue is a useful diagnostic feature in various diseases such as Kawasaki disease, TSS, scarlet fever, and group A streptococcal pharyngitis. In this article, we report chronological changes in strawberry tongue in TSST‐1–mediated exanthematous disease.

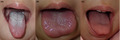

A 3‐year‐old girl presented to our hospital with acute onset of fever and rash. She was born by vaginal delivery at 37 weeks of gestation. She was previously healthy without any remarkable past medical history. Her vaccinations were up to date. Four days prior to the presentation, the dorsum of her left foot got burnt while playing with fireworks. On arrival at our hospital, she was lethargic. Her body temperature was 39.0°C. Although her blood pressure (94/52 mm Hg) was within the normal range, tachycardia (158/min) was noted. Physical examination revealed bulbar conjunctival injection and diffuse macular erythema. Prominent papillae covered by a white coating were observed on her tongue (white strawberry tongue) (Figure 1A). Although the patient did not meet the strict diagnostic criteria for toxic shock syndrome (TSS) proposed by the Centers for Disease Control and Prevention,^1^ she received a diagnosis of toxic shock syndrome toxin (TSST)‐1–mediated exanthematous disease related to *Staphylococcus aureus* infection.^2^ We initiated intravenous vancomycin and clindamycin treatment. Methicillin‐resistant *Staphylococcus aureus* (MRSA) with TSST‐1 production was isolated from the swab culture from her left foot. On the second hospital day, the patient became afebrile and diffuse erythema disappeared. On the third hospital day, the white coating desquamated, and red enlarged papillae gave the tongue a ripe strawberry look (Figure 1B). A set of blood cultures on admission was reported as negative. The patient's tongue appeared normal on the seventh hospital day (Figure 1C), and desquamation was noted on the extremities.

**Figure 1:**
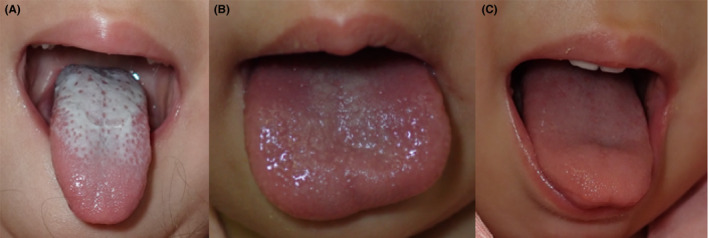
The dorsum of the tongue was covered with a white coating (white strawberry tongue) on admission (A). On the third hospital day, the white coating desquamated, and red enlarged papillae gave the tongue a ripe strawberry look (B). The papillae became less prominent and the tongue became less hyperemic on the seventh hospital day (C)

Strawberry tongue is a kind of enanthem of the tongue characterized by inflamed and hyperplastic fungiform papillae. In contrast, white strawberry tongue is the presence of a white coating on the tongue through which the hyperplastic fungiform papillae protrude. The white coating comprises keratinized epithelium of the filiform papillae.^3^ In patients with red strawberry tongue, the white coating desquamates, and a red and erythematous surface is denuded, interspaced with inflamed and hyperplastic fungiform papillae (similar to the seeds of a strawberry). Strawberry tongue can be a useful diagnostic feature in various diseases such as Kawasaki disease, TSS, scarlet fever, and group A streptococcal pharyngitis.^1^ TSST‐1–mediated exanthematous disease is a disease entity with signs and symptoms similar to those of TSS without hemodynamic disturbance.^2^ It is conceivable that TSST‐1–mediated exanthematous disease is a mild form of TSS or probable TSS proposed by Tofte et al.^4^ Blood cultures are rarely positive in staphylococcal TSS cases, in contrast to streptococcal TSS cases. Hence, it is reasonable the blood cultures were negative in our case. Strawberry tongue is occasionally observed in the early phase of scarlet fever, with red strawberry tongue occurring after 4–5 days.^5^ There are limited reports describing white strawberry tongue in TSS or TSST‐1–mediated exanthematous disease; the chronological changes in strawberry tongue are rarely reported. TSST‐1–mediated exanthematous disease and scarlet fever are bacterial toxin‐mediated diseases. Therefore, it is possible that the development of white strawberry tongue is associated with systemic reactions induced by bacterial toxins. Clinicians should pay attention to the morphological changes in the tongue to predict the pathogenesis of toxin‐mediated disease.

## CONFLICT OF INTEREST

The authors declare no conflicts of interest in association with the present study.

## AUTHOR CONTRIBUTIONS

A.S drafted the manuscript. Y.E, S.N, and Y.Y reviewed and edited the manuscript. All authors read and approved the final manuscript.

## ETHICAL APPROVAL

The informed consent was obtained from the patient's parents to publish this report (images in clinical medicine).
